# Promoting knowledge translation: An ecosystem approach to evidence in health

**DOI:** 10.1016/j.heliyon.2024.e28871

**Published:** 2024-03-29

**Authors:** Marcelle Miranda da Silva, Cristina Rosa Soares Lavareda Baixinho, Maria Fátima Mendes Marques, Claudia Sousa Oliveira, Renata de Moura Bubadué, Samhira Vieira Franco de Souza, Ivone Evangelista Cabral

**Affiliations:** a Escola de Enfermagem Ana Nery, Universidade Federal do Rio de Janeiro, 275 St Afonso Cavalcanti, Rio de Janeiro, RJ, Brazil; b Nursing Reserach, Innovation and Development Centre of Lisbon (CIDNUR), Av, Prof. Egas Moniz, 1600-190, Lisboa, Portugal; c Department of Rehabilitation of Nursing, Lisbon Nursing School, Av. Prof. Egas Moniz, 1600-190, Lisboa, Portugal; d Jean Piaget Higher School of Health, Jean Piaget Polytechnic Institute of the South, Jardim nº 1 do Enxerim, 8300-025, Silves, Portugal; e Universidade do Estado do Rio de Janeiro, 157 Blvd. 28 de Setembro, Rio de Janeiro, RJ, Brazil

**Keywords:** Evidence-based practice, Translational science, Biomedical, Health Personnel, Health knowledge, Attitudes, Practice, Health education

## Abstract

The dissemination and implementation of evidence in health contexts have been a concern of several international organizations responsible for recommending actions to health policymakers. World Health Organization has been advocating for an ecosystem of evidence to improve clinical practice and health professional education. Thus, in this article, we address the challenges to developing the evidence ecosystem from the point of view of health professional education, considering the contexts of practice and teaching, focused on knowledge translation. There are three pivotal challenges: producing qualified knowledge; adequate communication of the synthesized evidence; and institutional policy to sustain the implemented evidence in continuous and updated flow. The evidence ecosystem helps to understand these flows between the production and implementation of knowledge, based on the capacity and resources of different health systems. It needs to be developed in the field of health professional education, feedback in the contexts of practice and teaching, to contribute to third-generation knowledge being used by different users of health services.

## 1 Introduction

The dissemination and implementation of evidence in the health context have concerned several international organizations responsible for recommending actions for health policymakers. Organizations that systematize evidence synthesis include JBI®, Campbell, Cochrane, and the Canadian Institute of Health Research [1,2].

The World Health Organization (WHO) states that this set of evidence forms an ecosystem that reflects the formal and informal connections and interactions of different actors (and their capacities and resources) involved in the production, translation, and use of evidence [3]. In this sense, there is a collaborative interdependence to answer research questions that emerge from practice and maintain the cycles of essential knowledge for health care.

In health professional education, developing an evidence ecosystem can create cultures of care informed by evidence, starting from the basic educational process, with the student actively searching for synthesized knowledge, critically reading the available evidence, and applying it to clinical reasoning for decision-making.

The effectiveness of the evidence ecosystem is related to the generation, synthesis, translation and evaluation of knowledge in an environment that is both collaborative and competitive [4]. Therefore, the culture of evidence implementation requires the creation of an institutional atmosphere that encourages people towards the ontological belief that their experiences alone are not enough for qualified, safe and person-centered health care. Thus, they seek evidence, discuss their decisions with other professionals, and work toward the user's best interest.

The challenges for evidence-based clinical care require the initial understanding of evidence in its plural sense, “knowing that are not evidentiary in the sense of being "justified beliefs" substantiated by established systematic processes, but instead are based on claims for which the warrants for their truth value have been based on processes such as aesthetic knowing, personal knowing, or emancipatory knowing” [5:43–4].

So, there is more than one piece of evidence. It is essential to recognize that there is both tacit and scientific evidence [5]. Both types of evidence must go together in constant mutual criticism to promote transformations in practice and expansion of knowledge, because for the development of science it must be inseparable from professional education and practice [6].

Tacit evidence (or empirical, colloquial knowledge) is mainly informal. Usually, it includes opinions, values and habits of individuals with authorized discourse, such as policymakers, health professionals, users of health services, or citizens, expressed in different forms in formal deliberative dialogues, on electronic pages, in public policy documents, reports, or other formats. On the other hand, scientific or research evidence relates to explicit, systematic, and replicable or reproducible knowledge, subject to the judgment of its quality in terms of methodological standards and the stringency of its application [6].

To enable systematic implementation, scientific evidence should be translated and adapted to suit local work environments. This aligns with the principles of implementation science and quality improvement, which aim to achieve this adaptation effectively. Scientific evidence without human experience is meaningless.

However, the knowledge translation to clinical practice implies recognizing that there is not only one type of evidence and that the absence of sound scientific evidence should not generate immobility for the production of care since other factors are related to the implementation process, which include in addition to the best evidence, professional expertise, people's preferences, as in the person-centered care, and the availability of resources that imply viability and the flow of the workplace [6].

From the responsible perspective of recognizing the coexistence of evidence, we inform you that many terms describe the processes that apply knowledge into practice. We use knowledge translation, adopted by the Canadian Institute of Health Research, but to serve a broader audience, we present other ways of this term between different regions. “In the UK and Europe, the terms implementation science or research utilization are commonly seen in this context. In the USA, the terms dissemination and implementation, research use, knowledge transfer and uptake are often used” [6:14].

The knowledge translation approach is one of the most varied forms of transforming knowledge into action (KTA). In 2006, researchers [7] addressed the need for clarity in the conceptualization of KTA as a process that means “the exchange, synthesis and application of knowledge, produced under ethical conditions, translated by a complex system of interactions between researchers, decision-makers and users to accelerate the social use (action) of research benefits, with a view to improving the health of citizens; the creation of services, the use of more effective products and the strengthening of the health system” [8:15].

Thus, knowledge translation is an approach that contributes to third-generation knowledge to be used by different users of health services. Translating evidence into practice is a systematic process based on the pragmatism that using research results requires the professional to commit to ethics and accountability. Pragmatic and civic implementation science must be able to return the resources invested in science to promote collective well-being.

Identifying the need for knowledge, the production and availability of evidence-based care tools in the clinical field creates a virtuous circle of knowledge translation. Gaps in knowledge and the development of investigative and innovative competencies are why students and professionals only sometimes support decisions based on the best evidence [8].

Therefore, we will address the challenges for developing the evidence ecosystem from the point of view of health professional education, considering the contexts of practice and teaching, focused on knowledge translation.

## 2 Challenges of evidence-based clinical practice

Professional education is a process that occurs continuously throughout professional life, and is not limited to obtaining a bachelor's degree. However, upon completing the undergraduate program and entering the job market, it is common for professionals to stop seeking scientific evidence updates, which threatens the evidence ecosystem since the teaching-learning processes based on the student-professional relationship take place in practice environments.

This type of threat ends up being very serious because, unlike the scope of the Educational Health System, the evidence ecosystem is plural and diverse, referring to the entire landscape of evidence generation, synthesis, and dissemination across various sectors, including clinical practice. As it encompasses a wide range of disciplines and perspectives, the evidence ecosystem impacts critical and reflective professional action beyond the restricted use of evidence and data generated from clinical practice [9].

Although learning in research and the use of evidence requires clinical experience, and it is necessary to have contact with contexts concerned with the use of evidence to support decision-making, when we talk about the evidence ecosystem, we go beyond the use of evidence to support healthcare decisions patient individually. In this case, we incorporate into this person-centered care a collective decision-making process involving several interested stakeholders, assuming a prominent social and policy role.

We recognize that this chain of events comes from academia, which needs to do more to stimulate reflective critical thinking to deal with the multifaceted problems in practice. It is a concern of several educational and health institutions, which face difficulties in applying knowledge in clinical practice by incorporating linear models of knowledge translation to health contexts [10].

Sometimes, it is necessary to face the disparity between institutionalized operating procedures and new evidence that better informs care practices in clinical settings. In clinical practice, we often find routine care refractory to incorporating new evidence due to the lack of formal spaces that develop cultures of implementation science, that is, a culture of continual implementation of science in practice. Moreover, time gaps exist between the evidence produced/synthesized and its onset in the clinical field and the teaching-learning environments [11,12].

In this context, macro and micro-structural challenges stand out in applying evidence in clinical practice; however, we will focus on three pivotal challenges. Among the macro-structural challenges, the first is related to producing qualified knowledge. The best evidence requires that grounded knowledge comes from studies that apply meticulous, contextual, and epistemologically grounded research methodologies.

New knowledge only sometimes translates into behavior and organizational change, and significant gaps exist between the best practice and routine care. In health, research problems substantially emerge from practice contexts, and knowledge translation must be part of the research planning since the return of research results is one of the researcher's social accountabilities. One of the barriers to implementing knowledge in the clinical context is the erudition of scientific articles. In this sense, predicting the knowledge translation in the research project implies identifying the most appropriate language for the target audience and the type of knowledge to be translated.

Results of a scoping review reinforce that the technical and complex language used in scientific studies is an important difficulty in incorporating evidence into clinical practice. This makes it difficult for managers and the general public to understand and interpret evidence, which implies the necessary translation of scientific terms into a language that is accessible and understandable to everyone [13], based, for example, on the production of third-generation knowledge.

Hence, it is necessary to create strategies so that appropriate information reaches the hands of those who make health policies and are at the forefront of care. Thus, the second challenge corresponds to accessing the best synthesized evidence to establish implementation strategies in clinical care, weighing scientific evidence with tacit evidence. Conflicts of interest are part of this second challenge of implementing evidence whenever there are tensions between academic, commercial, financial, and ideological factors that can affect the specialists’ interpretation.

This second challenge is also highlighted in the aforementioned scoping review study, as it identifies in the literature that many managers and the general public do not have access to scientific journals or resources that summarize the evidence clearly. This lack of access makes it difficult to use evidence in decision-making and promoting health policies. In addition to the difficulty of access, this second challenge also involves assessing the quality of the evidence [13].

In this sense, professional education based on an ecosystem of evidence must support the ability to evaluate the quality of available information. It is necessary to promote education for critical analysis of evidence, as well as to transmit this evidence in a clear and accessible way through different communication strategies, which is related to plans for implementing third-generation generation knowledge [5].

The third and final challenge is the implemented evidence institutional policy of sustainability, maintaining a continuous and updated flow of access to the best evidence applicable to clinical practice. This challenge is associated with resistance to change commonly present in clinical practice contexts, both on the part of managers and the general public, and which may occur due to cultural barriers, lack of confidence in the evidence, lack of engagement, or adoption of practices traditionally deep-rooted [13].

These three challenges have become more evident since the beginning of the COVID-19 pandemic. Mapping the existing gaps between theory and practice amid the unknown allowed the identification and resolution of current problems concerning human care practices in COVID-19. For this purpose, the education of human capital, the survey of the necessary infrastructure, and the existing scientific evidence constitute the driving force for innovations, planned, agile, and timely decision-making, as in this health crisis.

Based on this example, we understand that it is up to the evidence ecosystem to contribute to the equity of options in global health. The necessary synthesis of evidence for the formulation and application of health policies, based on successful experiences, facilitates the identification of the different constrictions that prevent the implementation. The evidence ecosystem helps to understand these flows based on the capacity and resources of each health system.

## 3 Need to develop the evidence ecosystem for health professional education

It is necessary to take the evidence ecosystem as a highlight for education, which reflects on the teaching of creativity and the place of innovative knowledge in this process. The multifactorial characteristics lead to implementing innovations, especially in the global health context. Therefore, the multiple socioeconomic, political, and cultural realities between countries and the diversity of the theoretical-conceptual bases can guide the explanation of the effectiveness of implementing complex innovations in the health area. However, to be incorporated as new health care practices in a sustainable way, innovations require changes, adaptations, and effective coordination.

In the case of the Americas, despite underexamined knowledge translation initiatives, we highlight a Brazilian case that reasonably reflects the link between research and policy action by integrating the Evidence Informed Policy Network (EVIPNet) in the Americas, launched by the Pan American Health Organization/WHO, in 2007. "Brazil has led the way in implementing successful EVIPNet activities and products" [15:2].

Although it is not the only path for knowledge translation in the country, this network "published a series of eight health evidence syntheses for policy in key public priority areas", which sought to combat, for example, the high infant mortality rates in north and northeastern of Brazil, which was associated with poor quality of care during labor and delivery [15:2].

EVIPNet is an example of collaboration between researchers and clinicians that has helped Brazil and other countries in the Americas to face the challenges of knowledge translation, generating opportunities for capacity human resources, "assisting both researchers and decision makers through the network of the global EVIPNet initiative" [15:2].

Thus, the Brazilian commitment to the sectoral research policy and its interrelationship between the contexts of practice and teaching progressively reflected studies guided by the population's needs, which benefits from a universal public health system [14].

Developing the evidence ecosystem in the clinical field and professional health education reflect continuous feedback between research and public policy [15]. It also highlighted the need for a collaborative workforce in a partnership network. For that, we emphasize that teaching technical-scientific skills is as vital as teaching social and relational skills for health professionals to work collaboratively in a team. Moreover, in a virtuous cycle, the data produced in research emerge from practice and return to it in different ways: as social technologies, validated assessment instruments, instructional teaching materials, technological manuals, or protocols, among others.

Although there is an overlap between the concepts of the evidence ecosystem and the Learning Healthcare System, the latter emphasizes the practical application of evidence to improve patient outcomes. However, in this discussion, we focus on the evidence ecosystem, which encompasses a variety of related activities related to the generation and dissemination of evidence [16]. By understanding that the evidence ecosystem aims to facilitate the incorporation of updated knowledge into clinical practice, we confirm that developing clinical environments based on the Learning Healthcare System is also a challenge for this ecosystem.

We believe that the evolution of digital health and artificial intelligence will allow data to be continuously aggregated and analyzed in a complex environment for continuous production, dissemination, and use of scientific evidence in a collaborative and interconnected way, improving health care [16,17].

The development of scientific evidence in health professional education must be associated with strategies to create these products, which improve the systematic use of knowledge. The emphasis on the planning and creation of products, accompanied by explicit recommendations for their application and to facilitate knowledge translation, should be seen as an integral part of the use of knowledge beyond the synthesis of evidence [3].

Understanding that evidence can and should be presented according to the application context and target audience during the education process can encourage professionals who continually seek the best evidence to be producers of knowledge and articulate what is produced with practice.

Thus, the knowledge translation approach requires creative and innovative strategies with appropriate use of information. From this perspective, we open a space to discuss Open Science, configuring itself as a new paradigm for scientific communication, without distinction in society between “those who know” (experts) and those who “don't know” (stakeholders), in addition to providing transparency to the entire process of the scientific research cycle [18].

Open Science will contribute to knowledge without barriers within the evidence ecosystem, allowing scientific evidence to be accessible to all, regardless of geographic boundaries, academic and social status, and expanding the universe of creative knowledge, innovation, interculturality, and the closeness between consumers and producers of science.

With the involvement of users, the option for collaborative models of work between researchers and clinicians can accelerate this translation, thus being up to educational institutions to invest in teaching-service partnerships.

A recommended model for knowledge translation is the metatheoretical knowledge for action model, consisting of two articulated representative drawings: a funnel and a rotatory. The funnel of knowledge creation represents the funneling of three generations of knowledge (primary research and synthesis of available evidence) into a product or tool (the product of knowledge) with potential clinical applicability [7].

The rotatory of knowledge application or action cycle consists of seven steps: identification of the problem; evaluation and selection of knowledge; adaptation to the local context; assessment of barriers and facilitators; interventions on barriers with the selection, planning, and implementation; monitoring; evaluation of results; and sustainability of knowledge [7].

The exchange of knowledge, allowed by technological innovation, globalization, and knowledge translation, contributes to creating and implementing public policies. However, within this approach, it is worth highlighting the convergence between the evidence ecosystem, knowledge translation, and person-centered care. When evidence is translated into a more specific, robust, and organized field, individualized practices become stronger and meet the population's demand.

## 4 Conclusion

The evidence ecosystem supports understanding social contexts and addresses policy issues relevant to the knowledge that encourages innovation. It is necessary to value the consumption and the production of scientific evidence, to implement it into practice, as well as multidisciplinary work, user involvement in the health decision-making process, and the design of research projects that reflect real-life needs. In this sense, funding institutions must encourage student education through undergraduate, master's, and doctorate scholarships for the development of innovative research.

It is necessary to map the facilitators and barriers to communication and support of a health evidence ecosystem for knowledge translation. When person-centered, this knowledge can be more sustainable and universal, focusing on quality of life and equitable inclusion in education. After all, knowledge translated into a formal and informal context deals with the inclusion and autonomy of all social actors involved in the health care process, including students, health professionals and the population.

From the implications of the questions raised here, future developments in the field of knowledge translation, from the point of view of professional education in health, include the more active participation of academia to promote creative and innovative spaces to respond, through scientific research, to the needs of people, services, and systems. The education process must enable the student, within a favorable institutional policy of innovation, to foresee knowledge translation in their research project, affirming the ecosystem of evidence highlighted in education and practice in constant feedback.

## Data availability statement

No data was used for the research described in the article.

## CRediT authorship contribution statement

**Marcelle Miranda da Silva:** Conceptualization, Formal analysis, Funding acquisition, Investigation, Project administration, Supervision, Visualization, Writing – original draft, Writing – review & editing. **Cristina Rosa Soares Lavareda Baixinho:** Conceptualization, Formal analysis, Investigation, Visualization, Writing – original draft, Writing – review & editing. **Maria Fátima Mendes Marques:** Conceptualization, Formal analysis, Investigation, Visualization, Writing – original draft, Writing – review & editing. **Claudia Sousa Oliveira:** Conceptualization, Formal analysis, Investigation, Visualization, Writing – original draft, Writing – review & editing. **Renata de Moura Bubadué:** Conceptualization, Formal analysis, Investigation, Visualization, Writing – original draft, Writing – review & editing. **Samhira Vieira Franco de Souza:** Formal analysis, Visualization, Writing – original draft, Writing – review & editing. **Ivone Evangelista Cabral:** Conceptualization, Formal analysis, Investigation, Visualization, Writing – original draft, Writing – review & editing.

## Declaration of competing interest

The authors declare the following financial interests/personal relationships which may be considered as potential competing interests:Marcelle Miranda da Silva reports financial support was provided by Conselho Nacional de Desenvolvimento Científico e Tecnológico - CNPq (Process number: 200606/2022-0). If there are other authors, they declare that they have no known competing financial interests or personal relationships that could have appeared to influence the work reported in this paper.
